# *In Situ* Label-Free Study of Protein Adsorption on Nanoparticles

**DOI:** 10.1021/acs.jpcb.1c04775

**Published:** 2021-07-29

**Authors:** Christoph Bernhard, Marc-Jan van Zadel, Alexander Bunn, Mischa Bonn, Grazia Gonella

**Affiliations:** Max Planck Institute for Polymer Research, Ackermannweg 10, 55128 Mainz, Germany

## Abstract

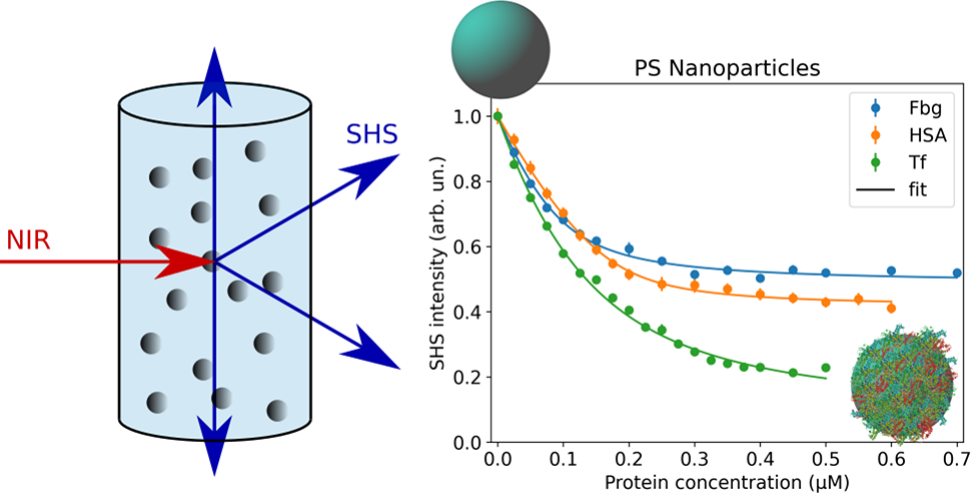

Improving the design of nanoparticles for use as drug carriers or biosensors requires a better understanding of the protein–nanoparticle interaction. Here, we present a new tool to investigate this interaction *in situ* and without additional labeling of the proteins and/or nanoparticles. By combining nonresonant second-harmonic light scattering with a modified Langmuir model, we show that it is possible to gain insight into the adsorption behavior of blood proteins, namely fibrinogen, human serum albumin, and transferrin, onto negatively charged polystyrene nanoparticles. The modified Langmuir model gives us access to the maximum amount of adsorbed protein, the apparent binding constant, and Gibbs free energy. Furthermore, we employ the method to investigate the influence of the nanoparticle size on the adsorption of human serum albumin and find that the amount of adsorbed protein increases more than the surface area per nanoparticle for larger diameters.

## Introduction

In the past years, the interaction of nanoparticles with proteins has been of broad scientific interest, particularly in the field of biomedical applications, where nanoparticles are commonly used as diagnostic agents and for drug delivery.^1−4^ In the case of drug nanocarriers, the fate of the nanoparticles within the human body is ultimately determined by their interaction with blood proteins.^5^ Therefore, further improvement of the drug nanocarrier design, for example, to enable site-specific targeting, requires a deeper understanding of the nanoparticle–protein interactions. So far, several different techniques, such as isothermal titration calorimetry, mass spectrometry, various spectroscopic methods, or SDS-PAGE, have been used to study nanoparticle–protein interactions.^3,6,7^ While these techniques have proven extremely useful in determining nanoparticle–protein interactions, they probe bulk behaviors and therefore cannot provide information on the interface or are only applicable *ex situ*. Naturally, one would like to investigate the interfacial properties of realistic nanoparticle systems and study the nanoparticle–protein interactions *in situ*. Second-order nonlinear optical techniques such as vibrational sum-frequency generation (SFG) spectroscopy or second-harmonic generation (SHG) are intrinsically surface-specific and, as such in principle, ideal techniques to investigate interactions at interfaces. Spectroscopic approaches have been extensively used to gain insight into the adsorption behavior, structure, and binding interactions of proteins on functionalized planar surfaces.^8−14^ However, because of the comparable size between the object of interest and the used wavelength of these optical techniques, it is impossible to simply apply these methods to investigate nanoparticle surfaces in the same way they are applied to planar surfaces.^15^ As in the case of their linear optical counterparts, this hurdle can be overcome by scattering methods, such as second-harmonic (SHS) or sum-frequency light scattering (SFS), which combine the surface specificity intrinsic to the nonlinear optical techniques with a scattering detection geometry and have been successfully employed to probe nanoparticle surfaces *in situ*.^15−19^

More specifically, SHS has been used in resonant and nonresonant conditions to characterize nanoparticle dispersions and the adsorption of molecular species. Resonant SHS relies either on resonances of the nanoparticles, such as, for example, the localized surface plasmon of metallic nanoparticles,^20−26^ or on (electronic) resonances of the adsorbate.^27−34^ Therefore, resonant SHS is necessarily limited in the types of nanoparticles and adsorbates that can be studied, given by the constraints of conventional laser wavelengths and can, in principle, suffer from photochemistry and two-photon fluorescence. On the other hand, nonresonant SHS, while lower in signal intensity as it is not enhanced by resonances, is not expected to influence the sample and provides a wider variety of potential applications. So far, nonresonant SHS has been used to study charged colloidal dispersions, nanodroplets, and screening of the surface charge.^35−42^

Here, we demonstrate a label-free nonresonant SHS method for *in situ* investigation of protein adsorption on nanoparticle surfaces. More specifically, we use nonresonant SHS to study the adsorption of blood proteins on negatively charged polystyrene (PS) nanoparticles with different surface functionalizations and sizes. By combining the experimental SHS data with a simplified adsorption model, namely a modified Langmuir model, we obtain information on the maximum amount of adsorbed protein per nanoparticle, the apparent binding constant, and consequently the apparent Gibbs free energy.

## Materials and Methods

The SHS setup used in this study is described in detail in the Supporting Information and is similar to the ones found in the literature.^15,43,44^ Briefly, the laser is focused in the center of a cuvette containing the colloidal solution. The generated second-harmonic signal is subsequently collected in the horizontal plane as a function of the scattering angle. To this end, the detection path is mounted on a rotation stage whose rotation axis coincides with the symmetry axis of the cuvette (see the Supporting Information for technical details). For the SHS experiments, we used commercial Polybead (Polysciences Europe, Germany) and further carboxylate-functionalized Polybead nanoparticles. The nanoparticle diameters were 100, 200, and 500 nm, respectively, and the stock dispersions of 2.5 wt % were diluted before use with ultrapure water (resistivity ∼18 MΩ). Sodium dodecyl sulfate (SDS) stabilized PS particles were synthesized according to the procedures in refs (45−47). The nanoparticle density for the SHS was adjusted to ∼4.55 · 10^11^ mL^–1^, ∼1.31 · 10^10^ mL^–1^, and ∼1.81 · 10^9^ mL^–1^, for the 100, 200, and 500 nm nanoparticles, respectively. Human serum albumin (HSA), human fibrinogen (Fbg), transferrin (Tf), and sodium chloride were purchased from Sigma-Aldrich (Germany) and used as received. Immunoglobulin G (IgG) was received from antibodies-online GmbH (Germany) and also used as received. The protein solutions were prepared in ultrapure water. After the addition of the protein solution, the nanoparticle dispersion was stirred with a magnetic stirrer for 1 min. Stirring was turned off before the SHS measurements. The samples were prepared in cylindrical quartz cuvettes (Hellma Analytics, Germany) with a diameter of 10 mm. The observed SHS signal intensity was solely generated by the nanoparticle dispersion in the focal volume, and the walls of the quartz cell did not contribute (see Figure S3 in the Supporting Information). All SHS measurements were performed at room temperature (22 ± 1 °C). The angle-resolved SHS scattering patterns were measured with an acceptance angle of ∼3.4 degrees. For the SHS titration experiments at a fixed detection angle, the acceptance angle was set to ∼13.5 degrees, and the polarization combination was set to p-in, p-out (*ppp*). The integration time for all measurements is 1 s, and each data point is averaged over a minimum of 10 measurements. All SHS titration experiments were performed on solutions in ultrapure water without additional sodium chloride.

We used a commercial dynamic light scattering (DLS) setup (Malvern, Zetasizer) to investigate particle sizes and agglomeration. The ζ-potential of the nanoparticles in a 1 mM sodium chloride solution was determined with a Malvern Zetasizer Nano-Z. The temperature for the DLS and ζ-potential measurements was 20 and 25 °C, respectively.

## Results and Discussion

### Theoretical Background

Nonresonant SHG and SHS have been intensively used in the past to investigate charged planar and nanoparticle surfaces.^35−39,48−53^ Commonly, second-order nonlinear optical processes are just considered to probe the interface. However, in the presence of charged moieties at the surface in contact with water, the electrostatic field generated at the surface leads to reorientation and polarization of water molecules. This leads to breaking of the centrosymmetry and therefore also generates a contribution to the second-harmonic response from bulk molecules. Thus, the detected second-harmonic intensity is considered to depend not only on a surface but also on a bulk contribution^40,50,54^
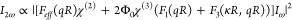
1where χ^(2)^ represents the susceptibility of interfacial molecules, whereas χ^(3)^ is the susceptibility from molecules in the bulk solution which are aligned and polarized due to the static electric field induced by the charges at the surface. Consequently, the described χ^(3)^ here contains also the χ^(2)^ contribution from the aligned/polarized water molecules.^55^*F*_*eff*_(*qR*), *F*_1_(*qR*), and *F*_3_(*κR*, *qR*) are scattering form factors: the former two depend on the scattering geometry and nature of the system (*R* is the nanoparticle radius and *q* is the scattering wave vector), while the latter also depends on the surface charge (κ is the inverse Debye length, with  where ϵ_0_, ϵ_*r*_, *k*_B_, *T*, *N*_A_, *z*, *e*, and *c* are the vacuum permittivity, relative permittivity of the solvent, Boltzmann constant, temperature, Avogadro’s number, valence of the symmetric electrolyte, elemental charge, and bulk electrolyte concentration, respectively) of the nanoparticles according to the nonlinear Rayleigh-Gans-Debye theory.^17,33,40,56,57^ Φ_0_ is the electrostatic surface potential. It has been found that the χ^(3)^ contribution, especially at low ionic strength, can significantly distort the scattering pattern.^40^ However, these form factors reduce to constants for a fixed scattering angle and ionic strength.

Ions in the bulk solution in contact with charged interfaces form an electrical double layer (EDL), which is generated by the balance between electrostatics, attracting the counterions close to the surface, and entropy, favoring counterions solvated in isotropic bulk conditions. In the case of spheres, and under the assumption of a diffuse EDL (low potential), the relationship between Φ_0_ and the surface charge density of a spherical nanoparticle σ_0_ can be approximated with an empirical formula^58^
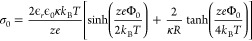
2which shows how the electric surface potential is intrinsically linked to the surface charge density, ionic strength, and temperature of the solution.

As a consequence, the nonresonant SHS, similar to the SHG signal for planar surfaces, is sensitive to changes in the surface charge density of the nanoparticle. In the present study, these changes are induced by the adsorption of (charged) proteins.

**Figure 1:**
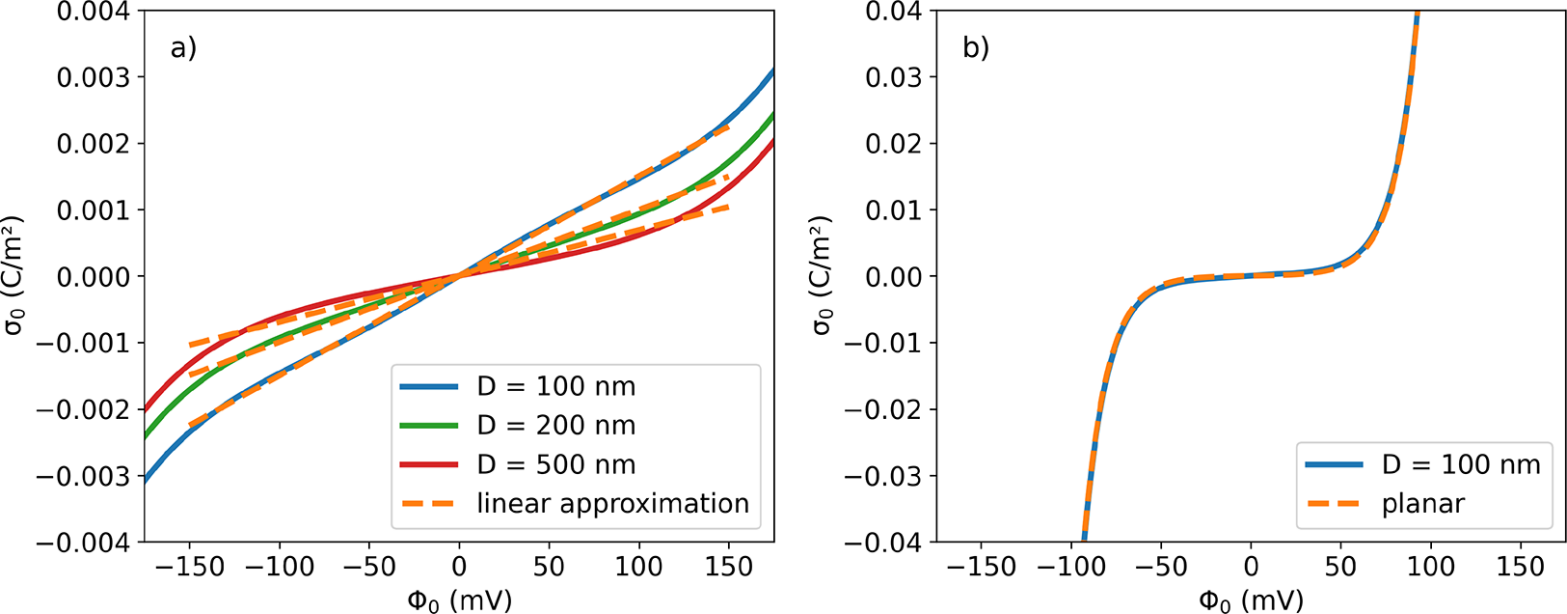
Surface charge density σ_0_ as a function of the surface potential Φ_0_ of the nanoparticles at a constant ionic strength of 1 μM: a) For a 1:1 electrolyte and particles of different diameters. Continuous lines are obtained from eq 2 for (blue) 100 nm, (green) 200 nm, and (red) 500 nm, respectively. The dashed orange lines show the respective linear approximation in the range from −150 to +150 mV. b) For a 4:4 electrolyte to model the net negative charge of HSA (about −8.0) at pH 6.6. Only the curve for *D* = 100 nm is shown for clarity, as the bigger diameter curves superimpose on it. The orange dashed line shows the function for planar surfaces for comparison.

Unfortunately, from eq 2 it is not possible to obtain an analytical inverse equation for Φ_0_ as a function of σ_0_. For relatively small particle sizes (*D* ≤ 100 nm) and under low ionic strength conditions (∼10^–6^ M for a 1:1 electrolyte), eq 2 shows a mostly linear behavior in the range from −150 to +150 mV for the surface potential, as shown in Figure 1.

For highly charged proteins or electrolytes with higher valence, such as a 4:4 electrolyte, for example, the behavior can nicely be approximated with the function for planar surfaces (see Figure 1b)
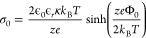
3which is easily invertible. This is also the case for large nanoparticles and/or high ionic strength conditions (see Figure S2 in the Supporting Information). For simplicity, in the remainder of this work, we will use the linear approximation shown in Figure 1 for the relation between Φ_0_ and σ_0_.

To describe the relative surface coverage θ_*cov*_ for a given protein concentration, we use a modified Langmuir adsorption model, which accounts for the depletion of the adsorbing species in bulk as described in ref (27). Strictly speaking, the simple Langmuir model cannot always describe the complex process of protein adsorption correctly.^59−61^ Nonetheless, the maximum amount of adsorbed protein can still be calculated from this simplified Langmuir model, whereas the binding constant can easily be underestimated.^59^ Despite that, owing to the simplicity of the model, we will use it as a starting point. According to ref (27), the relative surface coverage θ_*cov*_ is given by eq 4:
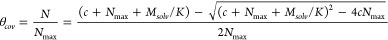
4

Here, *N*, *N*_max_, *M*_*solv*_, and *K* = *k*_*a*_/*k*_*d*_ are the amount of adsorbed proteins per unit volume, the maximum amount of adsorbed protein per unit volume, the molarity of the solvent (55.5 in the case of water), and the equilibrium binding constant, respectively. Through this simplified adsorption model, we gain access to the maximum amount of adsorbed protein, as well as the binding constant, from which we can calculate the Gibbs free energy (Δ*G*^0^)
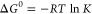
5where *R* is the ideal gas constant, and *T* is the absolute temperature. As mentioned above, the Langmuir model can result in quite large uncertainties of the binding constant and therefore the Gibbs free energy. Thus, in this work, we will refer to the obtained values from the fits as ”apparent” binding constant *K*_*app*_ and “apparent” Gibbs free energy .

### Charge Screening

**Figure 2:**
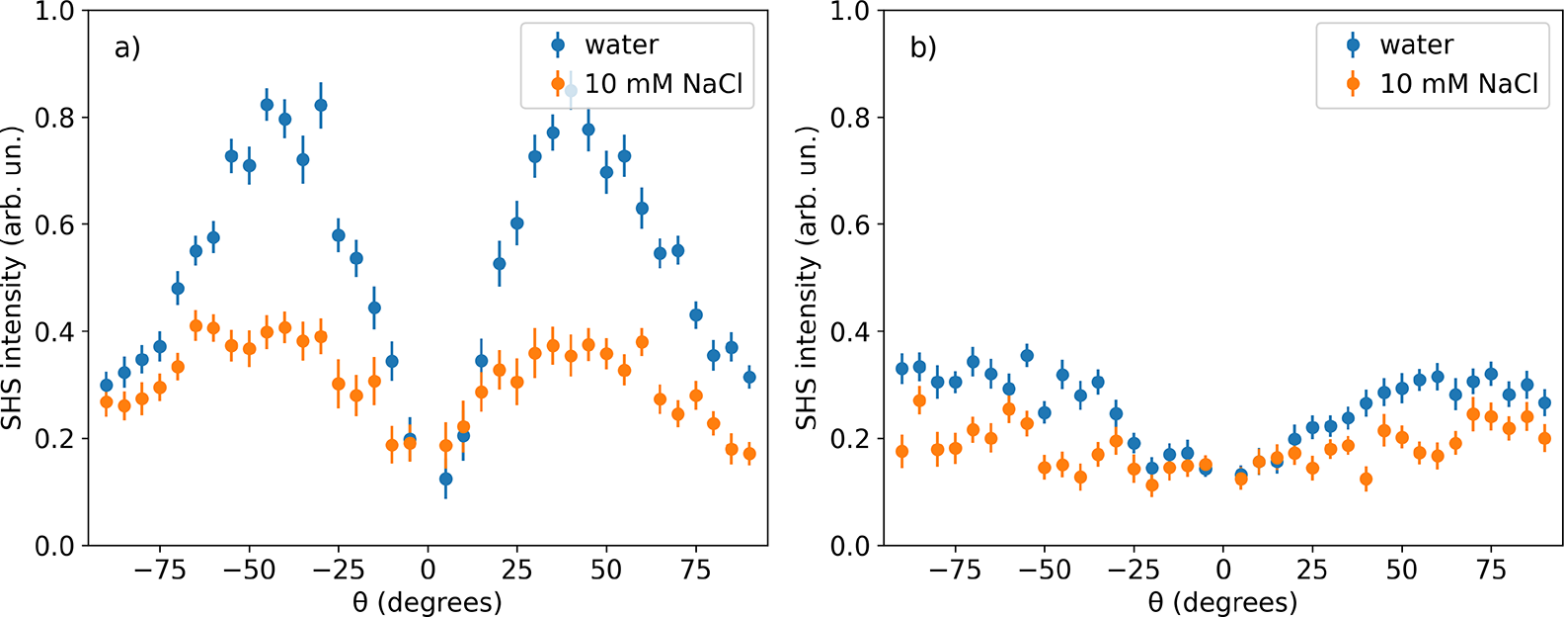
Angle-resolved nonresonant SHS pattern for SDS stabilized PS nanoparticles with a 100 nm diameter in a) *ppp* and b) *pss* (s-in, p-out) polarization combinations. The patterns were recorded for nanoparticles dispersed in ultrapure water (blue) and with 10 mM sodium chloride bulk concentration (orange).

First, we want to investigate the influence of charge screening on the SHS intensity and scattering pattern of PS nanoparticles. To this end, we measure the angle-resolved SHS pattern for sodium dodecyl sulfate (SDS) stabilized PS nanoparticles in ultrapure water and with 10 mM sodium chloride bulk concentration, as shown in Figure 2. The observed nonresonant SHS signal originates mainly from interfacial water molecules, which are aligned and/or polarized by the static electric field generated by the charges at the surface of the nanoparticles.^35,36,62^

The addition of 10 mM sodium chloride results in the screening of the surface charge and thus in the decrease of the modulus of the surface potential Φ_0_. Thus, according to eq 1, a decrease of the SHS signal intensity is expected. This is in agreement with the experimental observations for scattering patterns in both *ppp* and *pss* polarization combinations and for titration of sodium chloride in the bulk solution (see Figure S4 in the Supporting Information). Furthermore, from the angle-resolved SHS patterns in the *ppp* polarization combination, it is apparent that the decrease in signal intensity is more pronounced at scattering angles around 40 degrees than for 90 degrees, indicating that scattering angles close to the maximum intensity (∼40 degrees) are more sensitive to charge screening than larger scattering angles (∼90 degrees).

Additionally, it has been calculated in ref (40) that the maximum scattering intensity for PS nanoparticles with a 100 nm diameter shifts toward higher scattering angles when increasing the ionic strength. For instance, for a 1:1 electrolyte when increasing the concentration from 10^–5^ M to 10^–2^ M, the maximum moves toward higher angles by ∼25 degrees in *ppp* and ∼50 degrees in *pss*. Bigger nanoparticles, however, do not seem to be so sensitive, and this effect is even less pronounced for electrolytes with higher valence (see Figures S5 and S6 in the Supporting Information). As can be seen from Figure 2, the maximum SHS intensity in the *ppp* polarization combination for the nanoparticles in ultrapure water is detected at ∼40 degrees with respect to the incident fundamental light beam. This is at slightly larger scattering angles than that predicted from theory for low ionic strength.^40^ The discrepancy between the experimental results and theory could be due to residual SDS concentration in bulk, which can be already high enough to screen some of the signal contributions from the diffuse double layer.^40^ Similarly, also the maximum SHS intensity in the *pss* polarization combination is shifted toward higher scattering angles in the experiments as compared to the theory.

However, the addition of 10 mM sodium chloride into the bulk of the dispersion does not further shift the maximum SHS intensity toward higher scattering angles. These two findings, namely the increased sensitivity at 40 degrees scattering and no change in the shape of the scattering pattern, are important, as they can be exploited for SHS titration experiments at 40 degrees to increase the sensitivity, while not having to worry about possible changes in the scattering pattern shape due to the addition of proteins to the solution.

**Figure 3:**
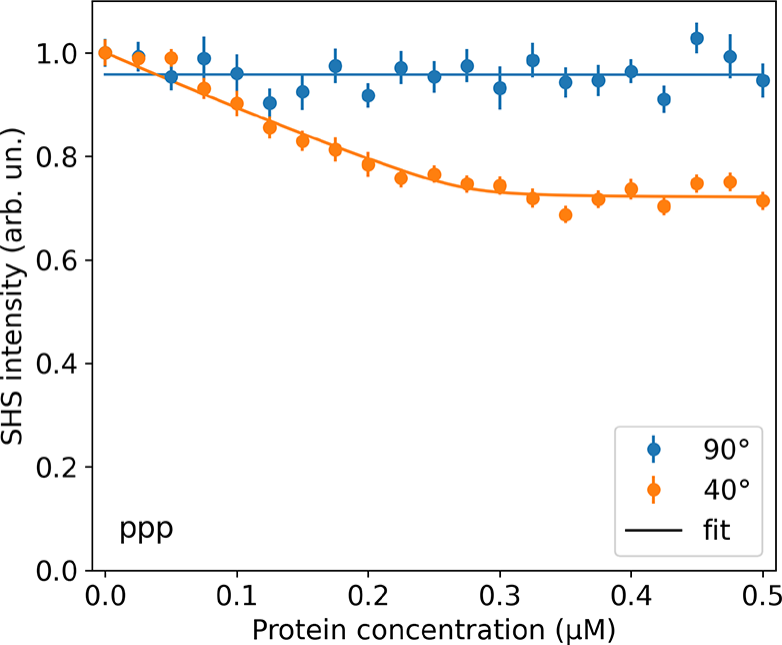
SHS intensity in the *ppp* polarization combination as a function of bulk Tf concentration for SDS-stabilized PS nanoparticles with a 100 nm diameter, detected at a (blue) 90 degrees and (orange) 40 degrees scattering angle. Solid lines represent fits to the measurements.

In the past, SHS titration experiments on gold nanoparticles, exploiting the resonant enhancement from the localized surface plasmon, have been performed at a fixed angle of 90 degrees.^25^ In general, this choice can lead to a signal that is not sensitive enough to observe protein adsorption, as shown for example in Figure 3. Here, no change in SHS intensity is observed for SDS stabilized PS nanoparticles upon adsorption of Tf at a 90 degrees detection, while the detected signal intensity at 40 degrees is clearly reduced. As a note, whenever a change of signal intensity at a 90 degrees scattering angle is observed, the data can be fitted using the same binding constant and saturation concentration as the measurements at 40 degrees detection (see Figure S7 in the Supporting Information). Consequently, the same information can be retrieved from measurements at either a 90 or 40 degrees scattering angle, but measurements at the scattering angle with maximum SHS intensity (∼40 degrees) have an increased sensitivity.

### Titration Experiments

In the next step, we performed SHS titration experiments to study the influence of the nanoparticle surface functionalization on the adsorption of various blood proteins.

**Figure 4:**
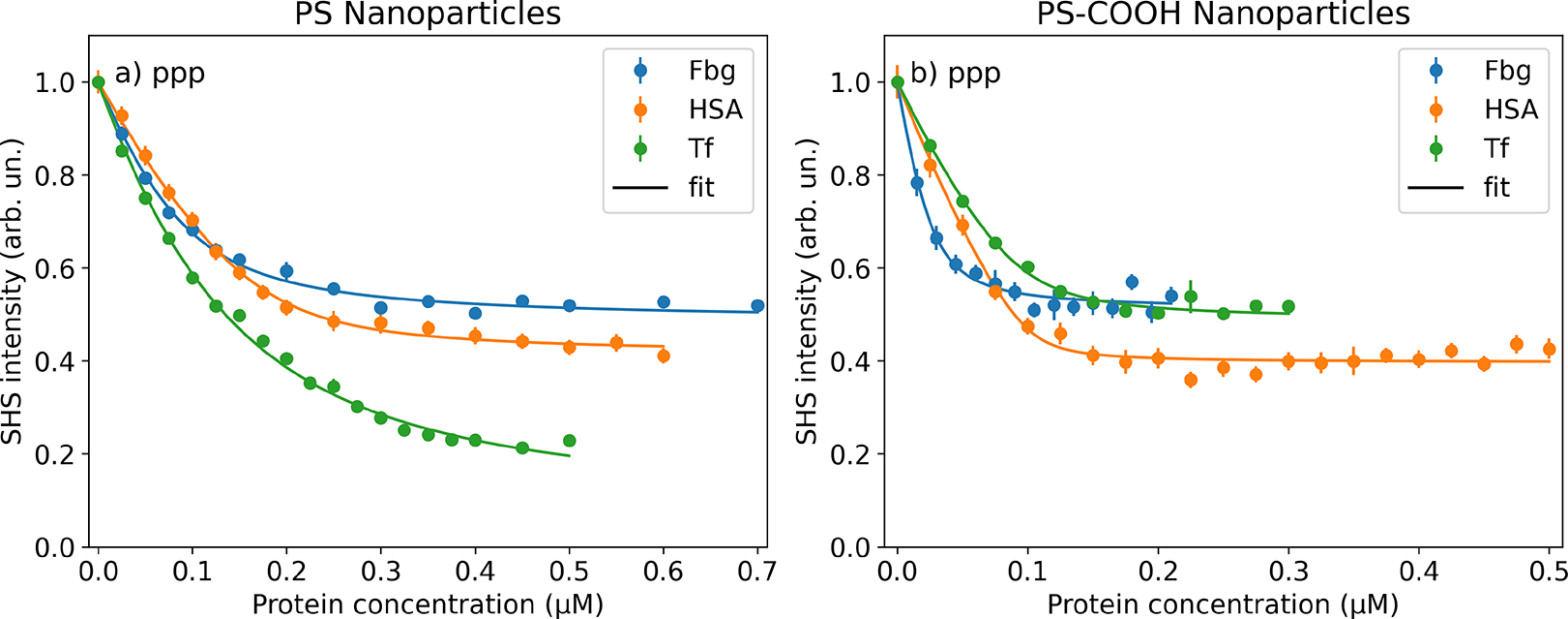
SHS intensity in the *ppp* polarization combination as a function of bulk (blue) Fbg, (orange) HSA, and (green) Tf concentrations for PS nanoparticles with a 100 nm diameter. a) Plain nanoparticles with a negative surface charge through residual sulfate ester from synthesis and b) with additional surface carboxyl groups.

Figure 4 shows representative results for the adsorption of Fbg, HSA, and Tf on plain PS nanoparticles and PS nanoparticles with carboxyl functionalization (PS-COOH). Both nanoparticle types carry an overall negative charge: the plain PS due to residual sulfate esters from synthesis and the PS-COOH due to deprotonation of the carboxylic groups at neutral pH. At pH values close to neutral, all three proteins also possess a net negative charge and therefore can still stabilize the nanoparticle dispersions upon adsorption. For both types of nanoparticles, the SHS intensity is reduced upon an increase of the protein concentration in bulk solution and can be described well by the modified Langmuir adsorption model. From the fit, it is possible to obtain the saturation concentration *N*_max_ and the apparent binding constant *K*_*app*_ for the individual proteins adsorbing on the respective nanoparticles. The results of the fits are summarized in Table 1. Interestingly, we observe a reduced SHS intensity upon adsorption of negatively charged proteins on negatively charged nanoparticles. This seems counterintuitive within the context of a mean-field theory, where the adsorption process is considered to be purely driven by electrostatics. However, in the case of proteins, the adsorption process is more complex, and protein adsorption on like-charged surfaces is widely observed.^25,45,63,64^ Especially for the interaction of polymeric nanoparticles with proteins, an enthalpy-driven adsorption process in combination with a reduction in entropy has been observed, which is contributed to mainly van der Waals, electrostatic, and hydrogen bond formation interactions.^7,45^ Furthermore, the reduced SHS signal intensity is in agreement with ζ-potential measurements, where a reduction of the modulus is measured after the adsorption of the proteins (e.g., from −42 ± 8 mV for the plain PS particles to −31 ± 8 mV after adsorption of HSA). Consequently, the modulus of the surface potential is reduced upon protein adsorption, even though no counterion condensation occurs. The fact that the SHS intensity is not reduced to zero is also in agreement with a residual surface charge after protein adsorption.

**Table 1:** Protein Adsorption Parameters Retrieved from the Fit of the SHS Experiments^a^

particle and protein	*K*_*app*_ (10^8^ mol^–1^)	*N*_max_ per particle	Δ*G*_*app*_^0^ (kJ/mol)
PS + Fbg	19 ± 5	130 ± 20	–(53.0 ± 0.7)
PS + HSA	33 ± 17	244 ± 20	–(54.4 ± 1.3)
PS + Tf	4.8 ± 1.4	183 ± 39	–(49.6 ± 0.7)
PS-COOH + Fbg	90 ± 50	38 ± 13	–(56.8 ± 1.4)
PS-COOH + HSA	270 ± 170	137 ± 8	–(59.6 ± 1.6)
PS-COOH + Tf	79 ± 30	131 ± 8	–(56.6 ± 0.9)

a The PS and PS-COOH nanoparticles have diameters of 100 nm and were dispersed in ultrapure water. The experiments were performed at fixed detection angles of 90 or 40 degrees.

Independently of the nature of the nanoparticle and the protein, we find for all measurements that the apparent Gibbs free energy is in the range from −50 to 60 kJ/mol, which is similar to what has been observed for protein adsorption on gold NPs and on planar silica surfaces.^25,48^ However, as mentioned in the theory section, one has to be careful with the interpretation of these apparent values, as the process of protein adsorption is in reality more complex than described by the Langmuir model.^59^ Still, the model can give useful insights concerning the maximum amount of adsorbed protein. Here, we observe differences between the individual proteins and nanoparticles. Similar amounts of Fbg and Tf adsorb on the plain PS nanoparticles, whereas roughly twice as much HSA adsorbs. For the PS-COOH nanoparticles, we observe a reduced maximum amount of adsorbed protein for all proteins. The plain and the carboxyl-functionalized nanoparticles possess very similar ζ-potentials of −(42 ± 8) and −(44 ± 11) mV, respectively. Therefore, this difference should not be induced by different surface charge densities. Tf is the least affected of the three proteins (*N*_max_ is roughly the same for the two different types of nanoparticles). Both Fbg and HSA adsorption are more strongly affected by the chemical nature of the surface, and the maximum number of adsorbed proteins reduces to roughly a third and half of the amount on the plain PS nanoparticles, respectively. The nanoparticle dispersions are stable upon addition of the proteins, as no aggregation is observed (see Figure S8 in the Supporting Information). However, if immunoglobulin G (IgG) is added to the plain nanoparticles, this leads to aggregation of the nanoparticles (see Figure S8 in the Supporting Information). This is probably because IgG has an isoelectric point of ∼7.5 and therefore is slightly positively charged at neutral pH. Upon adsorption, IgG would then neutralize the surface charge of the nanoparticles, which in turn leads to agglomeration of the nanoparticles. This neutralization of the surface charge does not occur for the negatively charged proteins, which prevents the aggregation of the nanoparticles.

**Figure 5:**
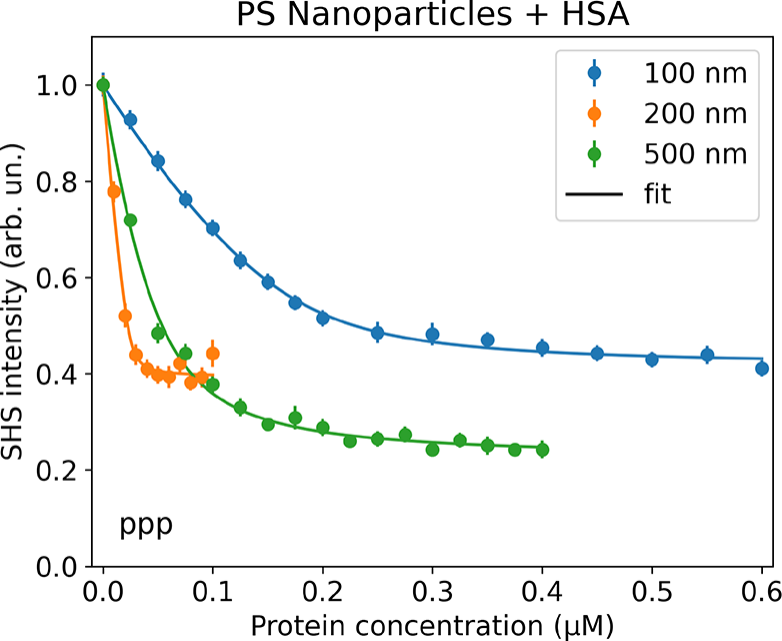
SHS intensity in the *ppp* polarization combination as a function of the bulk HSA concentration for PS nanoparticles with (blue) 100 nm, (orange) 200 nm, and (green) 500 nm diameters. Solid lines represent fits to the data.

Finally, we investigate how the nanoparticle size influences the adsorption of HSA. Figure 5 shows the SHS signal intensity as a function of bulk HSA concentration for plain PS nanoparticles with diameters of 100, 200, and 500 nm, respectively. The concentration-dependent SHS shows again a Langmuir-like behavior. The adsorption parameters retrieved from the fits are summarized in Table 2. Similar to the previous measurements, the apparent Gibbs free energy for HSA adsorption is again roughly in the range from −50 to 60 kJ/mol.

**Table 2:** Protein Adsorption Parameters Retrieved from the Global Fit of the SHS Titration Experiments^a^

particle diameter (nm)	*K*_*app*_ (10^9^ mol^–1^)	*N*_max_ per particle	Δ*G*_*app*_^0^ (kJ/mol)
100	3.3 ± 1.7	244 ± 20	–(54.4 ± 1.3)
200	80 ± 45	1179 ± 81	–(62.3 ± 1.4)
500	3.0 ± 0.8	18614 ± 2576	–(54.2 ± 0.6)

a The experiments were performed at fixed detection angles of 90 (for all particle sizes), as well as 40, 35, or 25 degrees for the 100, 200, and 500 nm nanoparticle, respectively.

With increasing the nanoparticle diameter, also the maximum amount of adsorbed HSA increases.

This is expected, as the available surface area per nanoparticle scales with the square of the diameter. However, with increasing the nanoparticle diameter, the mean area per HSA on the surface of the nanoparticles decreases from 129 nm^2^, over 106 nm^2^, to 42 nm^2^, indicating that the maximum amount of adsorbed protein increases more than the relative surface area per particle. Possible explanations for this could be differences in the surface charge densities of the nanoparticles and/or surface roughness variations. As discussed above, the surface potential of the nanoparticles is linked to their charge density. Therefore, we measured the ζ-potential of the nanoparticles to obtain an estimate of the surface potential. The ζ-potentials show a normal distribution centered around −42, – 50, and −54 mV for the 100, 200, and 500 nm nanoparticles with variances of 8, 7, and 8 mV, respectively. Assuming that the ζ-potential is similar to the surface potential of the nanoparticles, the resulting surface charge densities from small to large nanoparticle sizes are ∼−3.9, ∼−4.8, and ∼−5.3 mC/m^2^, respectively. Consequently, the surface charge density for the nanoparticles also increases with the nanoparticle size. This increase in surface charge density seems to favor HSA adsorption. Another possible explanation and additional contribution to the observed effect could be the curvature of the nanoparticles: The curvature of larger nanoparticles is lower as compared to smaller nanoparticles, and the lower curvature could favor HSA adsorption by enabling interaction with multiple binding sites.

## Conclusions

In conclusion, we have shown that nonresonant SHS can be successfully applied to observe protein adsorption on nanoparticles *in situ*, by simply exploiting the signal from the water molecules oriented by the field generated by the charges present at the surface of the nanoparticles and the subsequent screening effect of the protein adsorbing on the charged surface. Not relying on resonances, such as that of the localized surface plasmon of gold, this method has the potential for widespread applications, as most colloidal nanoparticles are charge-stabilized and would greatly benefit from an improved model that goes beyond the current limitation of the Langmuir adsorption model. The sensitivity of SHS titration experiments can be increased by performing them at a fixed detection angle close to the maximum intensity of the SHS scattering pattern. Furthermore, even by using a simplified model, such as the modified Langmuir model, it is possible to obtain insights on the protein adsorption and retrieve apparent binding constants and therefore the Gibbs free energy for the process. We find that the carboxyl functionalization of PS nanoparticles leads to a strong decrease in the amount of adsorbed Fbg and HSA, whereas it has only minor effects on Tf. Finally, from the results on the adsorption of HSA on PS nanoparticles with different sizes, we conclude that an increasing surface charge density in combination with a slightly reduced curvature favors the adsorption of HSA.

## Supporting Information Available

Detailed description of experimental SHS setup, data treament and further supplementary SHS and dynamic light scattering measurement data (PDF)
