# Supplementary material for: In Situ Label-Free Study of Protein Adsorption on Nanoparticles
Source: J Phys Chem B. 2021 Jul 29;125(31):9019–26. doi: 10.1021/acs.jpcb.1c04775 (PMC8389981; doi:10.1021/acs.jpcb.1c04775)
Supplement: Supplementary file 1 — jp1c04775_si_001.pdf [file jp1c04775_si_001.pdf]

Supporting Information: In-situ Label-free Study of Protein Adsorption on Nanoparticles

Christoph Bernhard,† Marc-Jan van Zadel,† Alexander Bunn,† Mischa Bonn,† and Grazia Gonella*,†,‡

†*Max Planck Institute for Polymer Research, Ackermannweg 10, 55128 Mainz, Germany*

‡*Current address: Institute of Biochemistry, Department of Biology, ETH Zürich, Switzerland*

E-mail: gonella@mpip-mainz.mpg.de

Experimental SHS Setup

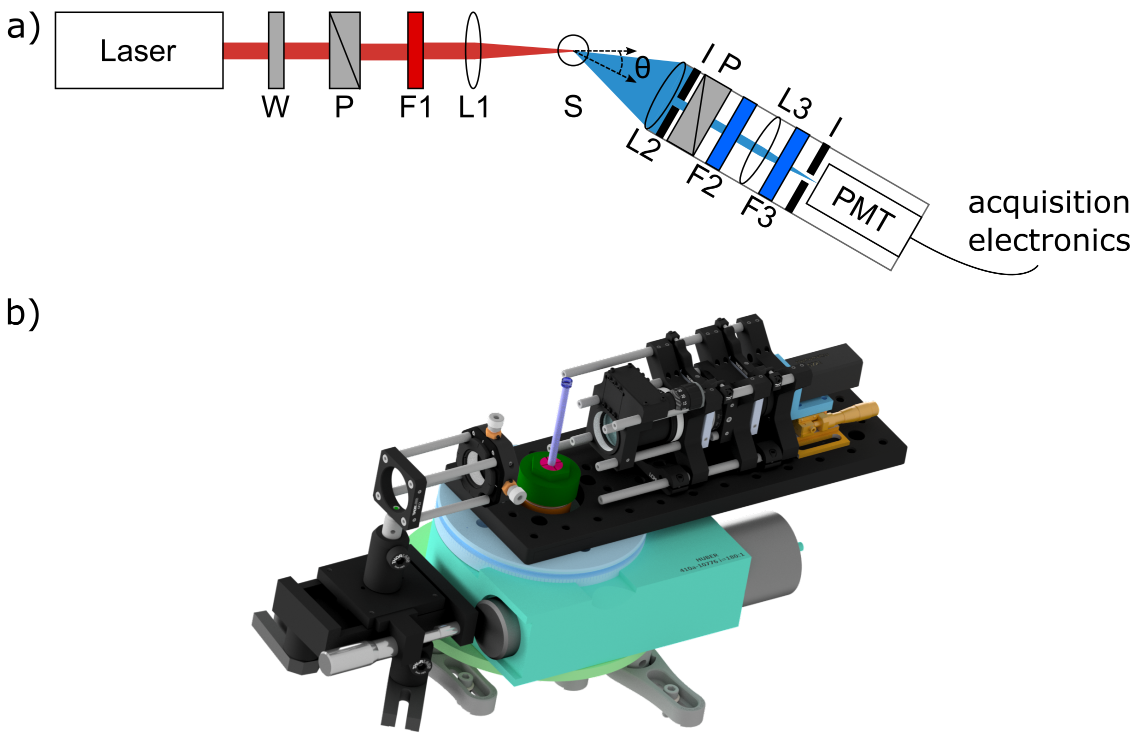

Figure S1: (a) Overview over the optical components of the SHS setup: (W) quarter-wave plate, (P) polarizer, (F1) long pass filter, (L1 and L3) focusing lens, (L2) collimation lens, (F2 and F3) bandpass filters, (I) iris diaphragm. The fundamental beam is shown in red, scattered second harmonic light in blue. (b) 3D image of the SHS setup showing the sample and the SH detection path located on a rotational goniometer stage.

The SHS setup described here is similar to the ones in Refs. S1–S3. Figure S1 shows a scheme of the experimental scattering setup. The source for the fundamental laser beam at (1028 ± 5) nm is a mode-locked Yb:KGW (ytterbium-doped potassium gadolinium tungstate) laser (Pharos-15W, Light Conversion, Lithuania) with a tunable repetition rate and output power. Throughout the experiments in this work, the repetition rate is set to 1 MHz, and the fundamental beam power is set in the range of 80-150 mW before the sample. The polarization of the fundamental beam is controlled with a Glan-Taylor polarizer (GT10-B, Thorlabs, USA). Beforehand the fundamental beam is circularly polarized by a quarter-wave plate (AQWP10M-980, Thorlabs, USA), so that the laser power after the polarizer is independent of the polarization state. A long pass filter (FEL850, Thorlabs, USA) filters

out higher harmonic frequency contributions from the laser. Afterward, the beam is focused with a plano-convex lens (f = 7.5 cm) in the sample cell. We use cylindrical cuvettes (10 mm diameter, Hellma Analytics, Germany) for the SHS and DLS measurements. The beam waist in the focus is ~33 μm with a corresponding Rayleigh length of ~0.83 mm. A collimation lens (f = 5.0 cm) collects the generated scattered SH light from the sample. An iris diaphragm and a Glan-Taylor polarizer (GT10-A, Thorlabs, USA) are used to determine the solid angle and the polarization over which the signal is integrated. The acceptance angle in this work is set to ~3.4 degrees for angle-dependent SHS measurements, while it is ~13.5 degrees for SHS experiments at a constant detection angle. Two bandpass filters (ET525/50, Chroma, USA and FGB39, Thorlabs, USA) filter out the fundamental laser beam before detection. Finally, the signal is focused onto a photomultiplier tube (PMT, H10721-20, Hamamatsu, Japan). The generated electrical pulse from the PMT is further amplified (HFAC-26dB, Becker & Hickl, Germany) and sent to a time-correlated single photon counting board (TimeHarp Nano 260, Picoquant, Germany). To improve the signal to noise ratio, the signal is manually gated in the measurement software with a gate width of ~12.5 ns. The detection path of the setup is mounted on a rotational goniometer stage (410A, Huber Diffraktionstechnik, Germany), which enables the collection of scattering patterns in the horizontal plane from −110 degrees to +110 degrees, where 0 is the direction of the fundamental.

SHS: Data Treatment and General Assumptions

The particle surface is not the sole signal source which is detected, but other sources could contribute to the emission at 2ω: the particle bulk, the HRS from the solvent and that from the molecule/protein of interest. Thus, we measure for each sample the emission at 2ω from the bare NPs at the concentration of interest. We also measure mole dependently the HRS from the molecules/proteins and that from the solvent. For our specific systems under study, it turned out that no signal can be detected from the proteins in solution without particles and thus these two contributions will be disregarded for the rest of this work. To correct

for the incoherent HRS contributions, the obtained data are normalized in two ways: First, the HRS from the pure solvent is subtracted from the sample signal. Afterward, the sample signal is divided by the HRS signal of bulk water in *sss* polarization combination, to correct for angular differences of the focal volume. Thus, the normalized SH signal $SHS_{pii}(\theta)$ is obtained via equation 1:

$$SHS_{pii}(\theta) = \frac{I_{pii}(\theta) - HRS_{S,pii}(\theta)}{HRS_{H_2O,sss}(\theta)} \quad (1)$$

Here, ii represents the incoming polarization (either p or s), $I_{pii}(\theta)$ is the p-polarized intensity generated by the sample, $HRS_{S,pii}(\theta)$ the p-polarized HRS intensity of the bulk solution in absence of particles and $HRS_{H_2O,sss}(\theta)$ is the HRS signal of water in *sss* polarization combination.

In addition, for all SHS measurements performed in this study, we apply the following assumptions for the data treatment:

a) The particle density is low enough so that multiple scattering is negligible. This assumption was verified experimentally for the 100 nm PS particles (data not shown).

b) Liquids are spatially isotropic and only produce incoherent HRS.

c) We consider the medium of our samples as lossless and dispersion-free. This means that Kleimann symmetry can be applied to the samples of interest.[S4]

The Influence of Particle Diameter and Ionic Strength on the Surface Charge Density of Spheres

As shown in figure S2, the empirical function for spheres to describe the surface charge density dependent on the surface potential converges to the one for planar surfaces for high salt electrolyte concentrations or increasing nanoparticle diameter.

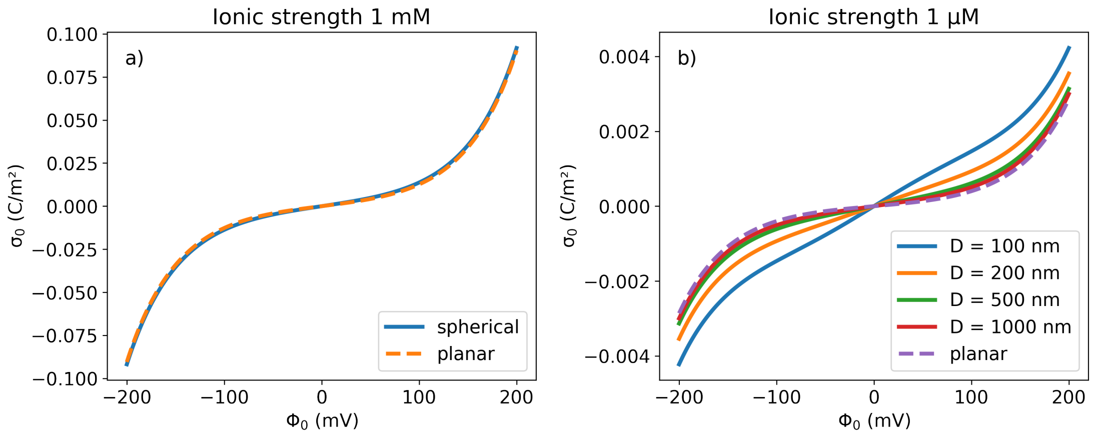

Figure S2: The surface charge density σ_0 as a function of the surface potential Φ_0 of the nanoparticles in comparison to the behavior at planar interfaces. a) For a 1:1 electrolyte with bulk concentration of 10^{-3} M and a nanoparticle diameter of $D = 100$ nm. b) For a 1:1 electrolyte with bulk concentration of 10^{-6} M and varying nanoparticle diameter.

SHS: Signal Contributions

The walls of the quartz cuvette which is used for the SHS experiments, do not contribute to the detected SHS signal intensity, as shown in figure S3. The detected signal intensity for the empty cuvette is comparable to the dark counts of the detector. Upon addition of water and the nanoparticles, both the signal shape and intensity are strongly changed from the bulk HRS signal. Upon further addition of PS nanoparticles the signal intensity and shape is further changed.

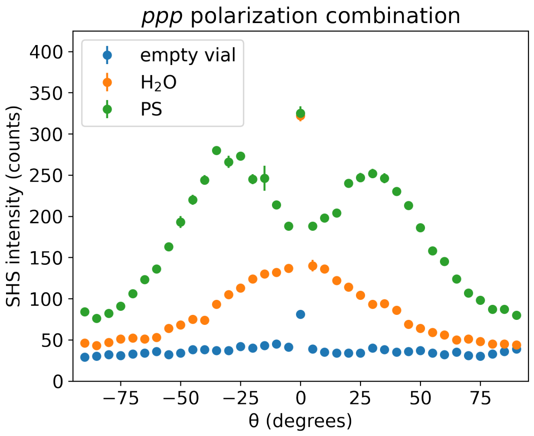

Figure S3: Angle-resolved non-resonant SHS pattern for a) the empty Hellma cuvette (blue), b) the cuvette filled with ultrapure water (orange), and c) after addition of PS nanoparticles with a diameter of 100 nm (green).

SHS: Charge Screening

Figure S4 shows the SHS signal intensity from a SDS-stabilized PS nanoparticle dispersion in water as a function of the sodium chloride bulk concentration. The detected SHS signal intensity decreases upon screening of the nanoparticle surface charge due to the addition of sodium chloride.

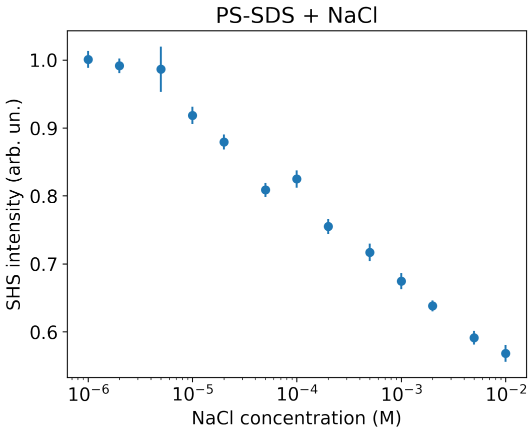

Figure S4: SHS intensity as a function of bulk sodium chloride concentration for SDS stabilized PS nanoparticles with 100 nm diameter detected at 40 degrees scattering angle.

Theoretical Angle-resolved SHS Patterns

The scattering patterns in figures S5 and S6 were calculated according to Ref. S5. For small nanoparticles with a diameter of 100 nm, the maximum is shifted towards higher scattering angles upon increase of the electrolyte concentration. This effect is less pronounced for nanoparticles with a larger diameter of 1000 nm. Here, the shape of the scattering patterns is almost unchanged with increasing electrolyte concentration.

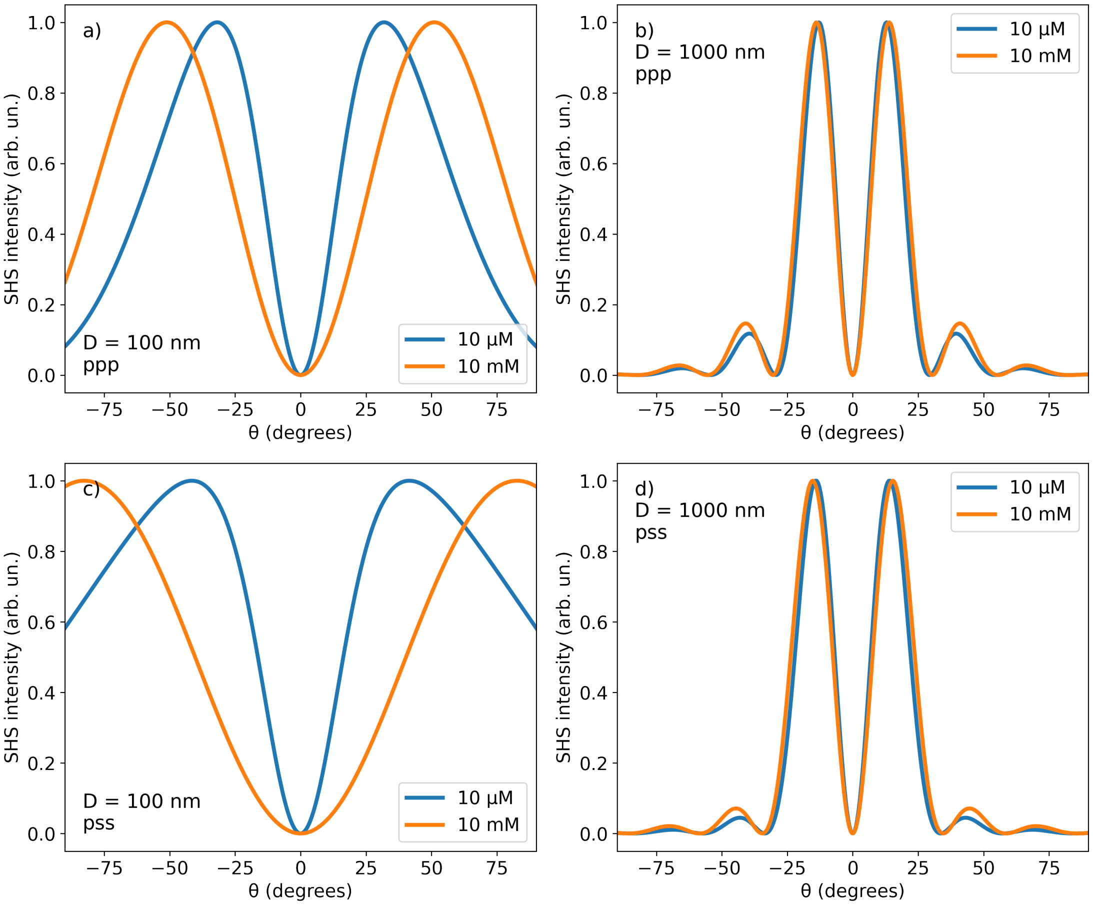

Figure S5: Calculated scattering patterns for a 1:1 electrolyte with (blue) 10^{-5} M and (orange) 10^{-2} M concentration in a-b) *ppp* and c-d) *pss* polarization combination for particles with a,c) 100 nm and b,d) 1000 nm diameter.

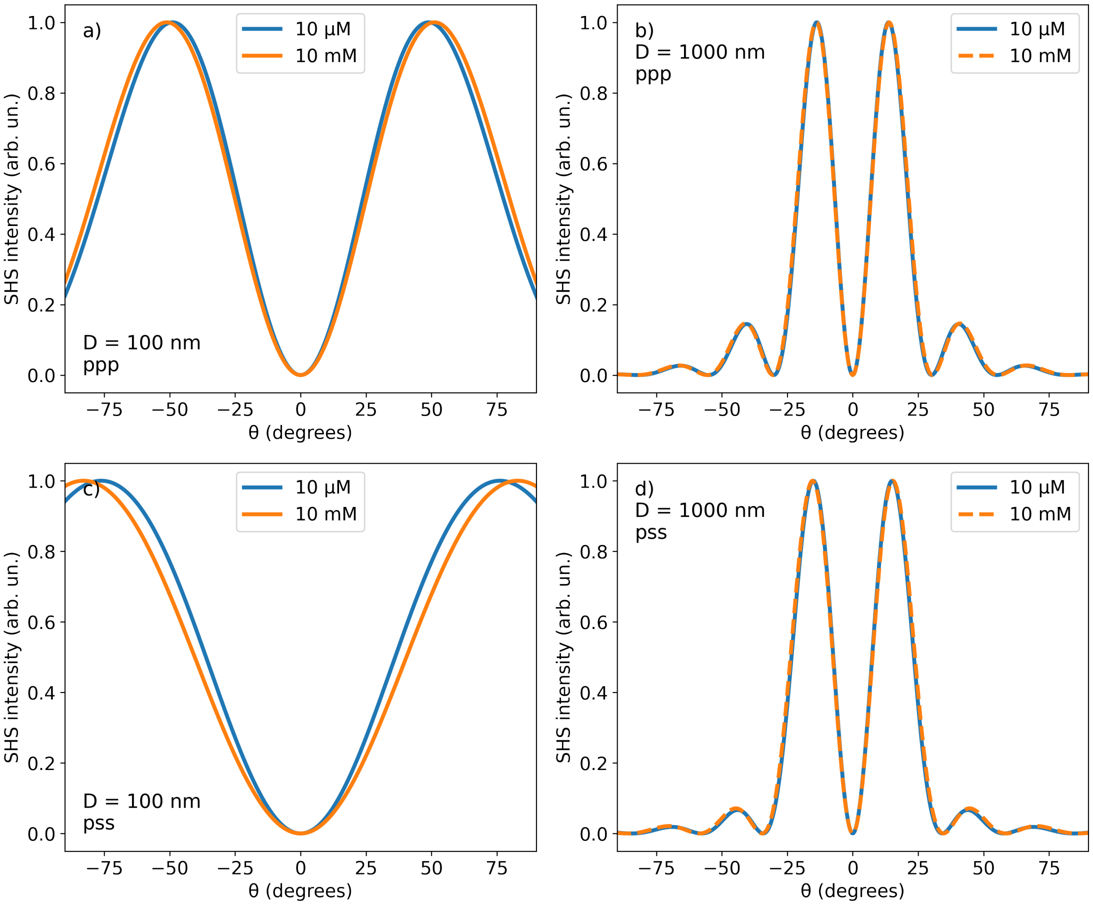

Figure S6: Calculated scattering patterns for a 4:4 electrolyte with (blue) 10^{-5} M and (orange) 10^{-2} M concentration in a-b) *ppp* and c-d) *pss* polarization combination for particles with a,c) 100 nm and b,d) 1000 nm diameter.

For 4:4 electrolytes almost no change in the shape of the scattering patterns is observed even for smaller nanoparticles with with a diameter of 100 nm. For *pss* polarization combination a shift can still be observed, but less strong as in the case of a 1:1 electrolyte in figure S5. Furthermore, no change in the shape of the scattering patterns in both *ppp* and *pss* polarization combination can be observed for larger nanoparticles with a diameter of 1000 nm.

Angle-dependent SHS Sensitivity

Figure S7 shows the SHS intensity as a function of bulk TF concentration for 100 nm diameter PS nanoparticles for detection at both 90 and 40 degrees with respect to the propagation of the fundamental beam. The experimental data can be nicely fitted using the same binding constant and maximum amount of adsorbed protein and they only differ in the amount the initial SHS signal is reduced, as indicated by the different scaling factors A in table S1.

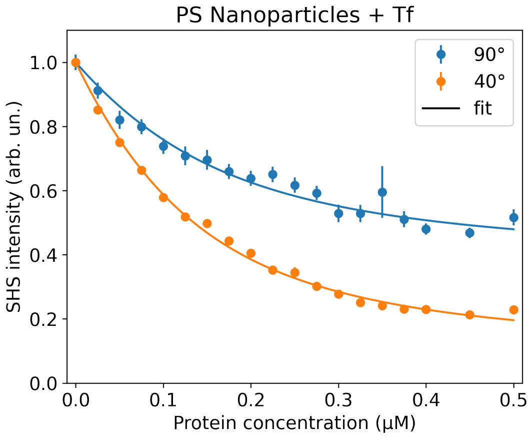

Figure S7: SHS intensity as a function of bulk Tf concentration for PS nanoparticles with 100 nm diameter, detected at (blue) 90 degrees and (orange) 40 degrees scattering angle. Solid lines represent fits to the measurements using the same binding constant and maximum amount of protein per unit volume in the fit.

Table S1: Tf adsorption parameters on PS nanoparticles with 100 nm diameter retrieved from a global fit of the SHS titration experiments. The experiments were performed at fixed detection angles of 90 and 40 degrees for the different nanoparticle sizes.

Scattering angle (degrees)	A	K_{app} (10^8 mol^{-1})	N_{max} (10^{-7} mol)
90	0.40 ± 0.03	5.0 ± 1.6	1.4 ± 0.3
40	0.72 ± 0.04		

Dynamic Light Scattering

Figure S8 shows the determined hydrodynamic diameter from DLS measurements of the pure nanoparticles in water, and after the addition of either HSA or IgG in the solution. The pure nanoparticle dispersion shows a narrow distribution around 100 nm, as expected. Upon addition of HSA in the solution, almost no change is observed. However, the addition of IgG in the solution induces a shift toward bigger hydrodynamic diameter and an additional peak at around 5 μm appears, indicating agglomeration of the dispersion. This agglomeration upon addition of IgG is also visible to the eye, while this is not the case for all the other proteins.

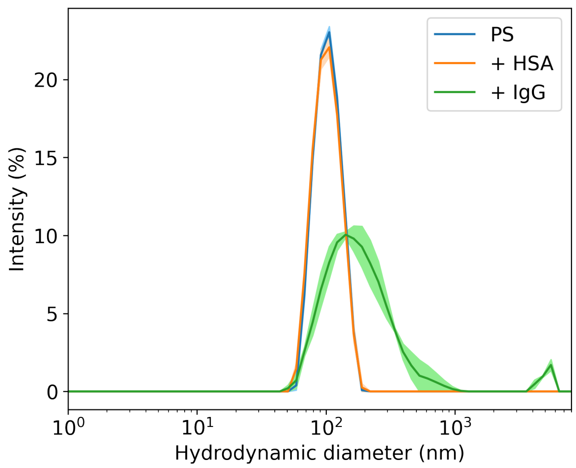

Figure S8: Hydrodynamic diameter of the plain 100 nm PS nanoparticles in water (blue) and after addition of either (orange) 300 nM HSA or (green) 150 nM IgG. Shading around the solid lines represents uncertainty of the data based on 3 measurements.

References

(S1) Gomopoulos, N.; Lütgebaucks, C.; Sun, Q.; Macias-Romero, C.; Roke, S. Label-free second harmonic and hyper Rayleigh scattering with high efficiency. *Optics Express* **2013**, *21*, 815.

(S2) Lütgebaucks, C. U. Lipid membrane characterization with second harmonic scattering: surface potentials, ionization, membrane asymmetry and hydration. Ph.D. thesis, École Polytechnique Fédérale de Lausanne, 2017.

(S3) Roke, S.; Gonella, G. Nonlinear Light Scattering and Spectroscopy of Particles and Droplets in Liquids. *Annual Review of Physical Chemistry* **2012**, *63*, 353–378.

(S4) Boyd, R. W. *Nonlinear Optics*, 3rd ed.; Academic Press/Elsevier: Amsterdam, 2008.

(S5) Gonella, G.; Lütgebaucks, C.; de Beer, A. G. F.; Roke, S. Second Harmonic and Sum-Frequency Generation from Aqueous Interfaces Is Modulated by Interference. *The Journal of Physical Chemistry C* **2016**, *120*, 9165–9173.
